# Supplementary material for: THINGSvision: A Python Toolbox for Streamlining the Extraction of Activations From Deep Neural Networks
Source: Front Neuroinform. 2021 Sep 22;15:679838. doi: 10.3389/fninf.2021.679838 (PMC8494008; doi:10.3389/fninf.2021.679838)
Supplement: Supplementary file 1 [file Data_Sheet_1.pdf]

In the code example below, we demonstrate both the flexibility and ease of use of our toolbox compared to using solely PyTorch. THINGSvision is more versatile and does not require the user to be adept in PyTorch, whereas when using PyTorch more knowledge about neural network architectures and tensor dimensions is crucial. This might not cause difficulties for someone experienced in Python programming and Machine Learning, but is not trivial for researchers who are not as familiar with this area of Computer Science.

THINGSvision

```
import torch
import thingsvision.vision as vision

from thingsvision.model_class import Model

device = 'cuda' if torch.cuda.is_available() else 'cpu'

model = Model(
              model_name='vgg16',
              pretrained=True,
              model_path=None,
              device=device,
              backend='pt',
              )


dl = vision.load_dl(
                    root=IMG_PATH,
                    out_path='.\tests`,
                    batch_size=64,
                    transforms=model.get_transformations(),
                    backend='pt',
                    )



features, targets = model.extract_features(
                                            data_loader=dl,
                                            module_name='features.10',
                                            batch_size=64,
                                            flatten_acts=True,
                                            device=device,
                                            )


vision.save_features(features, `.\test`, `.npy`)
```

PyTorch

```
import os
import torch

import numpy as np
import torchvision.models as models

from torch.utils import DataLoader, TensorDataset
from torchvision import datasets, transforms as T


device = torch.device(`cuda` if torch.cuda.is_available() else `cpu`)
model = models.vgg16()
model.to(device)
model.eval()
normalize = transforms.Normalize(mean=[0.485, 0.456, 0.406], std=[0.229, 0.224, 0.225])
transforms = T.Compose([T.Resize(256), T.CenterCrop(224), T.ToTensor(), normalize])


# Note: since loading images into memory, converting them into the correct format,
# and subsequently creating a dataset involves more lines of code
# and additional imports, we use pseudo-code for this part

# load in images ...

(e.g., tuple(torch.from_numpy(skimage.io.imread(...)) for ... in os.listdir(IMG_PATH)
if ... img.endswith(...))

# create a dataset ...

(e.g., TensorDataset(...))

data_loader = DataLoader(dataset, batch_size=32, shuffle=False)

# Note: we register a forward hook as it is the most elegant and versatile
# way to store activations of a neural network model, and is recommended by PyTorch.
# We know, however, that there are other ways to do this, such as simply truncating
# the model at the point of extraction, but we deem registering a forward hook superior.

module_name = `classifier.3`

def get_activation(name):
    """Store hidden unit activations at each layer of model."""
    def hook(model, input, output):
        try:
            activations[name] = output.detach()
        except AttributeError:
            activations[name] = output
    return hook


def register_hook(model):
    """Register a forward hook to store activations."""
    for n, m in model.named_modules():
        m.register_forward_hook(get_activation(n))
    return model
```

```python
# initialise dictionary to store hidden unit activations on the fly
global activations
activations = {}
# register forward hook to store activations
model = register_hook(model)

# Note: it is not feasible to use the entire dataset as an input to the model,
# which is why we have to iteratively process subsamples of the data

features = []
with torch.no_grad():
    for batch in data_loader:
        batch = batch.to(device)
        out = model(batch)
        act = activations[module_name]
        features.append(act)

features = np.asarray(features)

with open(os.path.join(OUT_PATH, `features.npy`, `rb`) as f:
    np.save(f, features)

# Note: be aware of the fact that above code will not work
# for every neural network architecture and every layer,
# and does neither involve computing nor storing target vectors
# or softmax probabilities which may be of interest to the user
# (e.g., if the desired module is an early layer of the model,
# and the format should be .txt, more lines of code are necessary)
```
